# Hospital‐ and county‐level characteristics explain geographic variability in prices of cancer‐related procedures: Implications for policy and interventions

**DOI:** 10.1002/cam4.6792

**Published:** 2023-12-22

**Authors:** Jennifer L. Moss, Savanna G. Ledford, Veronica Bernacchi, Chan Shen

**Affiliations:** ^1^ Department of Family and Community Medicine Penn State College of Medicine, The Pennsylvania State University Hershey Pennsylvania USA; ^2^ Department of Public Health Sciences Penn State College of Medicine, The Pennsylvania State University Hershey Pennsylvania USA; ^3^ Department of Surgery Penn State College of Medicine, The Pennsylvania State University Hershey Pennsylvania USA

**Keywords:** geography, healthcare costs, metropolitan status, social determinants of health

## Abstract

**Background:**

Healthcare costs in the U.S. are high and variable, which can hinder access and impact health outcomes across communities. This study examined hospital‐ and county‐level characteristics to identify factors that explain geographic variation in prices for four cancer‐related procedures.

**Methods:**

Data sources included Turquoise Health, which compiles publicly‐available price data from U.S. hospitals. We examined list prices for four procedures: abdominal ultrasound, diagnostic colonoscopy, brain MRI, and pelvis CT scan, which we linked to characteristics of hospitals (e.g., number of beds) and counties (e.g., metropolitan status). We used multilevel linear regression models to assess multivariable relationships between prices and hospital‐ and county‐level characteristics. Supplementary analyses repeated these models using procedures prices for commercial insurance plans.

**Results:**

For each procedure, list prices varied across counties (intraclass correlation: abdominal ultrasound = 23.2%; colonoscopy = 17.1%; brain MRI = 37.2%; pelvis CT = 50.9%). List prices for each procedure were associated with hospital ownership (all *p* < 0.001) and percent of population without health insurance (all *p* < 0.05). For example, list prices for abdominal ultrasound were higher for proprietary versus Government‐owned hospitals (*β* = 539.10, 95% confidence interval [CI]: 256.12, 822.08, *p* < 0.001) and for hospitals in counties with more uninsured residents (*β* = 23.44, 95% CI: 2.55, 44.33, *p* = 0.03). Commercial insurance prices were negatively associated with metropolitan status.

**Conclusions:**

Prices for cancer‐related healthcare procedures varied substantially, with considerable heterogeneity associated with county location as well as county‐level social determinants of health (e.g., health insurance coverage). Interventions and policy changes are needed to alleviate the financial burden of cancer care among patients, including geographic variation in prices for cancer‐related procedures.

## 1 INTRODUCTION

In the United States, geographic variation in healthcare pricing and spending is evident.^1^ For a given procedure, pricing can differ by a factor of 10 or more.^2^, ^3^ These variations are related to costs needed to provide services in a specific area (e.g., local rent^1^ and wages^4^), facility‐specific factors (e.g., academic status),^3^ negotiations between insurers and providers, and patient health status.^1^ Prices account for 70% of geographic differences in healthcare spending.^4^ Across counties, market competition is a key determinant of pricing because providers in low‐access counties (e.g., counties with fewer providers or health insurance plans^5^; rural counties^6^) can negotiate higher reimbursement rates, resulting in higher prices.

These variations have real implications for healthcare access, utilization, and debt among patients, particularly cancer patients. Half of U.S. adults report foregoing healthcare due to costs,^7^ and high healthcare prices disproportionately impact patients with low socioeconomic status and patients without health insurance.^7^ Healthcare spending associated with cancer diagnosis and treatment has rapidly increased in recent years, resulting in “financial toxicity” which can cause adverse effects such as stress and financial hardship.^8^, ^9^ In response to these concerns, the Centers for Medicare and Medicaid Services (CMS) implemented a new rule on January 1, 2021, mandating that hospitals disclose their pricing to empower patients, enhance market competition, and curtail healthcare costs.^10^, ^11^

Despite the high burden of healthcare costs, gaps remain in our understanding of geographic variation in prices of cancer‐related healthcare procedures. In this study, we leveraged data from 2004 U.S. hospitals to characterize geographic variability in pricing for four procedures that may be used during cancer diagnosis and treatment (abdominal ultrasound, diagnostic colonoscopy, magnetic resonance imaging [MRI] scan of the brain, and pelvis computerized tomography [CT] scan with contrast). These findings provide insight into geographic differences in cancer‐related healthcare pricing, with implications for access and accessibility of care among cancer patients. Future research can leverage these findings to inform price transparency interventions^3^ or policies^10^ to reduce healthcare pricing, spending, and debt.

## 2 METHODS

### 2.1 Data sources and measures

#### 2.1.1 Hospital‐level outcome variables: Procedure prices

We extracted pricing data from Turquoise Health.^12^ Turquoise Health compiles publicly‐available, machine‐readable pricing data from U.S. hospitals (in compliance with the CMS Hospital Price Transparency Regulation), including separate prices by health insurance types/plans. The current analysis focused on hospitals' “list price” for each procedure, as well as supplementary price measures for commercial insurance plans.

The four cancer‐related healthcare procedures we investigated were defined using Current Procedural Terminology (CPT) codes: Abdominal ultrasound (CPT code: 76700); diagnostic colonoscopy (CPT code: 45378); brain MRI (CPT code: 70553); and pelvis CT scan (CPT code: 72193).

#### 2.1.2 Hospital‐level independent variables

Turquoise Health compiles a number of hospital‐specific variables, including ownership (government, physician, proprietary, or volunteer [i.e., charity]), type (acute care or critical access), compliance score (range: 1–5, with higher scores indicating greater compliance), and number of beds (range: 1–2891).^12^ Hospital ownership types have become more complex in recent decades, with many blended models emerging; however, in general, these systems can be considered not‐for‐profit (i.e., government and volunteer/charity) or for‐profit (i.e., physician‐owned or proprietary [investor‐owned]).^13^ Compliance scores reflect the degree to which hospitals comply with the CMS price disclosure rules.^12^

#### 2.1.3 County‐level independent variables

We collected county‐level variables from 2020 Area Health Resource File^14^: Census region (Midwest, Northeast, South, or West),^15^ metropolitan status (metropolitan/urban or non‐metropolitan/rural),^16^ county population (in 1000s), and density of primary care physicians (PCP; number of PCPs per 100,000 population). We also gathered indicators of social determinants of health: percent without health insurance, percent living below the federal poverty level, percent unemployed (among population ages 16+ in the civilian workforce), and percent with a bachelor's degree (among population ages 25+ years). In addition, we collected life expectancy from 2020 County Health Rankings (CHR)^17^ to capture general healthfulness of the population.

We geocoded each hospital's address and linked hospital‐ and county‐level data using a federal information processing system (FIPS) code.^18^ Multiple locations within a health system were treated as separate hospitals. Our dataset included 2004 hospitals that reported prices for at least one procedure. These hospitals represented 1319 out of 3143 (42.0%) U.S. counties (average hospitals per county = 1.8; range: 1–29).

## Statistical analysis

First, we generated descriptive statistics for the independent variables, i.e., frequency and proportion for categorical variables and median, mean, and 95% confidence interval (CI) for continuous variables.

Then, we generated the median, mean, 95% CI, and coefficient of variation (CV) for the list price of each procedure. We generated choropleth maps to depict variability in prices across counties; for counties with more than one price for a procedure, we calculated the arithmetic mean of the prices within that county.

Next, we used multilevel linear regression models^19^, ^20^, ^21^ to assess the relationships between independent variables and the list price for each procedure, using hospital as the unit of analysis. Separately for each procedure, we ran an “empty model” to calculate the intraclass correlation (ICC),^21^ which summarizes the degree of clustering in prices for hospitals nested within counties (Model 0). Then, we regressed the list price for each procedure on hospital variables (level‐1 variables; Model 1) and county variables (level‐2 variables; Model 2). Finally, we regressed the list price on all of the hospital‐ and county‐level variables (“full model”; Model 3). Supplementary analyses repeated these procedures for commercial insurance prices; for parsimony, we report the findings for Models 0 and 3 for the supplementary analyses.

Statistical analyses used a two‐sided *p*‐value of 0.05. Analyses were conducted using SAS version 9.4 (Cary, NC). Per U.S. federal regulations, this analysis was exempt from ethics review because it did not involve human subjects. Informed consent was not obtained because all data were aggregated at the hospital or county level (i.e., no participant interaction was involved in the study).

## 3 RESULTS

Most hospitals (*n* = 2004) were classified as volunteer ownership (70.9%) and acute care (79.5%), with a mean of 200 beds (Table S1). The counties in which they were located (*n* = 1319) were primarily in the South (42.6%) or Midwest (36.2%). On average, counties had 60.6 PCPs per 100,000 population (95% CI: 58.6–62.6), 10.8% of the population without health insurance (95% CI: 10.5–11.0), and estimated life expectancy of 77.8 years (95% CI: 77.6–77.9).

**FIGURE 1:**
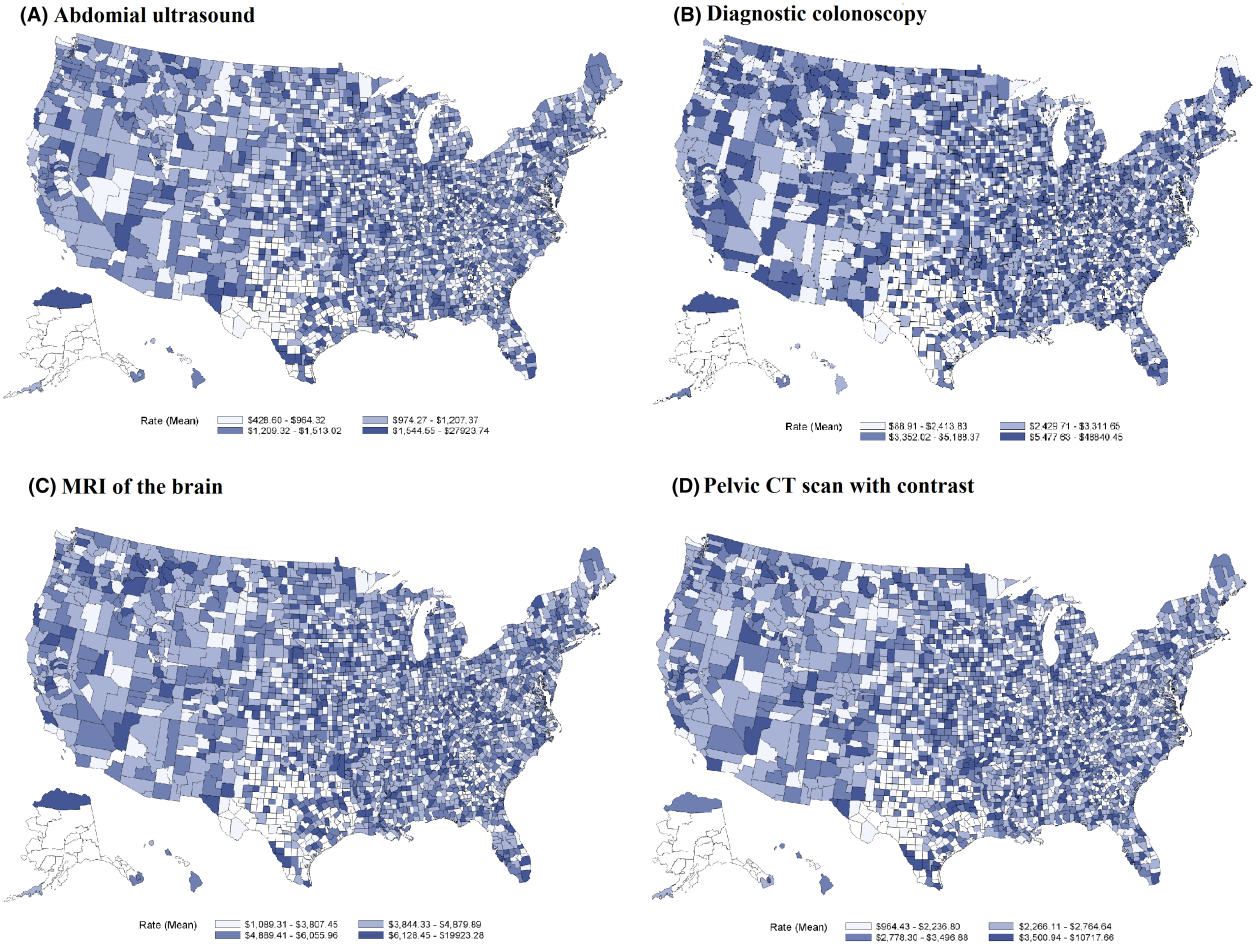
County‐level average of hospital list prices for (A) abdominal ultrasound, (B) diagnostic colonoscopy, (C) magnetic resonance imaging (MRI) of the brain, and (D) pelvis computerized tomography (CT) scan with contrast.

The mean list price was $1267 (95% CI: 1200–1335) for abdominal ultrasound, $4780 (95% CI: 4185–5375) for diagnostic colonoscopy, $4988 (95% CI: 4832–5144) for brain MRI, and $2883 (95% CI: 2787–2979) for pelvis CT scan (Table S2; Figure 1). Compared to the list prices, prices for commercial insurance tended to be lower (e.g., the mean price for an abdominal ultrasound was $1267 for list price vs. $669 for commercial insurance).

### 3.1 Multilevel characteristics and prices for abdominal ultrasound

In Model 0 (the empty model estimating clustering in prices), the ICC for abdominal ultrasound prices indicated that 23.2% of the variation in prices across hospitals was attributable to county location. In Model 1 (assessing the relationships between prices and hospital‐level variables), prices differed by hospital ownership and type (both *p* < 0.01). In Model 2 (assessing the relationships between prices and county‐level variables), prices differed by Census region, county population, and percent uninsured (all *p* < 0.05). In Model 3 (the full model assessing the relationships between prices and hospital‐ and county‐level variables), prices were higher for hospitals that were proprietary versus government ownership (*β* = 539.10, 95% CI: 256.12, 822.08, *p* < 0.001) and lower for critical access versus acute care hospitals (*β* = −355.11, 95% CI: −573.81, −136.41, *p* < 0.01) (Table 1). In addition, prices were higher for hospitals in counties in the South versus Midwest region (*β* = 266.47, 95% CI: 24.68, 508.26, *p* = 0.03) and with higher percent uninsured (*β* = 23.44, 95% CI: 2.55, 44.33, *p* = 0.03). The ICC for the Model 3 was 13.6%, indicating that these variables accounted for 41.2% of the variation in prices.

**TABLE 1:** Multilevel relationships between hospital‐ and county‐level characteristics and hospital list prices for cancer‐related procedures.

	Abdominal ultrasound			Diagnostic colonoscopy			MRI of the brain			Pelvic CT scan with contrast		
	(*n* = 1396)			(*n* = 862)			(*n* = 1360)			(*n* = 1331)		
	Beta	95% CI		Beta	95% CI		Beta	95% CI		Beta	95% CI	
*Level 1: Hospital‐level characteristics*												
Ownership												
Government	(ref)			(ref)			(ref)			(ref)		
Physician	−66.30	(−991.54, 858.94)		2450.97	(−5273.12, 10175.06)		978.04	(−764.62, 2720.7)		839.98	(−368.24, 2048.20)	
Proprietary	539.10	(256.12, 822.08)	***	7226.66	(4965.58, 9487.74)	***	2428.90	(1885.92, 2971.88)	***	1483.30	(1158.51, 1808.09)	***
Volunteer	163.15	(−73.74, 400.04)		1503.73	(−372.46, 3379.92)		710.72	(248.10, 1173.34)	**	410.38	(135.29, 685.47)	**
Type												
Acute care	(ref)			(ref)			(ref)			(ref)		
Critical access	−355.11	(−573.81, −136.41)	**	−366.21	(−2258, 1525.58)		−159.50	(−598.46, 279.46)		−227.14	(−485.68, 31.40)	
Compliance score	18.24	(−52.67, 89.14)		−353.67	(−960.31, 252.97)		−22.83	(−161.95, 116.30)		−8.82	(−92.19, 74.54)	
Number of beds (100 s)	15.98	(−20.89, 52.86)		402.07	(84.14, 720.00)	*	88.90	(19.29, 158.50)	*	48.32	(6.20, 90.43)	*
*Level 2: County‐level characteristics*												
Census region												
Midwest	(ref)			(ref)			(ref)			(ref)		
Northeast	44.32	(−229.2, 317.84)		−587.42	(−2957.98, 1783.14)		287.87	(−272.30, 848.04)		−87.06	(−435.13, 261.02)	
South	266.47	(24.68, 508.26)	*	536.37	(−1481.78, 2554.52)		857.42	(367.64, 1347.20)	***	320.85	(20.68, 621.02)	*
West	27.66	(−250.89, 306.22)		597.36	(−1572.87, 2767.59)		828.05	(258.40, 1397.70)	**	250.62	(−101.10, 602.34)	
Metropolitan status												
Non‐metropolitan/Rural	(ref)			(ref)			(ref)			(ref)		
Metropolitan/Urban	−126.85	(−324.18, 70.48)		437.31	(−1197.6, 2072.22)		−305.70	(−701.01, 89.61)		−59.44	(−301.67, 182.80)	
County population (1000s)	0.06	(−0.03, 0.15)		0.53	(−0.09, 1.16)		0.12	(−0.09, 0.34)		0.05	(−0.10, 0.20)	
Density of PCPs (number/100,000 population)	−0.70	(−3.66, 2.26)		−6.12	(−30.11, 17.87)		−3.69	(−9.63, 2.24)		−1.21	(−4.94, 2.53)	
% without health insurance	23.44	(2.55, 44.33)	*	232.77	(60.62, 404.92)	**	54.36	(11.36, 97.37)	*	98.51	(72.08, 124.94)	***
% below the federal poverty level	−5.45	(−17.17, 6.27)		34.68	(−59.82, 129.18)		7.07	(−15.34, 29.48)		−8.34	(−21.42, 4.73)	
% unemployed^a^	6.57	(−5.43, 18.57)		−45.83	(−142.86, 51.20)		21.47	(−1.98, 44.93)		22.78	(8.90, 36.65)	**
% with bachelor's degree^b^	−2.91	(−9.84, 4.02)		−21.53	(−79.14, 36.07)		6.77	(−6.49, 20.03)		3.59	(−4.19, 11.36)	
Estimated life expectancy (years)	24.32	(−12.73, 61.37)		212.99	(−94.83, 520.81)		108.57	(32.09, 185.05)	**	86.03	(39.91, 132.16)	***
ICC	13.6%			8.4%			27.6%			41.5%		

Abbreviations: CI, confidence interval; ref, reference; PCP, primary care provider.

^a^ Among population, ages 16+, in the civilian workforce.

^b^ Among adults 25+ years.

* *p* < 0.05;

** *p* < 0.01;

*** *p* < 0.001.

### 3.2 Multilevel characteristics and prices for diagnostic colonoscopy

In Model 0, the ICC for diagnostic colonoscopy was 17.1%. Prices differed by hospital ownership and number of beds (Model 1) as well as county‐level population and percent uninsured (Model 2; all *p* < 0.05). In Model 3, prices were higher for hospitals that were proprietary versus government ownership (*β* = 7226.66, 95% CI: 4965.58, 9487.74, *p* < 0.001) and with more beds (*β* = 402.07 per 100 beds, 95% CI: 84.14, 720.00, *p* = 0.01), and for hospitals in counties with higher percent uninsured (*β* = 232.77, 95% CI: 60.62, 404.92, *p* = 0.01) (Table 1). The ICC for Model 3 was 8.4%, indicating that these variables accounted for 51.0% of the variation in prices.

### 3.3 Multilevel characteristics and prices for brain MRI

In Model 0, the ICC for brain MRI was 37.2%. Prices differed by hospital ownership and number of beds (Model 1) as well as county‐level Census region, population, and percent uninsured (Model 2; all *p* < 0.05). In Model 3, prices were higher for hospitals that were proprietary or volunteer versus government ownership (proprietary: *β* = 2428.90, 95% CI: 1885.92, 2971.88, *p* < 0.0001; volunteer: *β* = 710.72, 95% CI: 248.10, 1173.34, *p* < 0.01) and with more beds (*β* = 88.90, 95% CI: 19.29, 158.50, *p* = 0.01), and for hospitals in counties in the South or West versus Midwest region (South: *β* = 857.42, 95% CI: 367.64, 1347.20, *p* < 0.001; West: *β* = 828.05, 95% CI: 258.40, 1397.70, *p* < 0.01), with higher percent uninsured (*β* = 54.36, 95% CI: 11.36, 97.37, *p* = 0.01), and with higher life expectancy (*β* = 108.57 per year, 95% CI: 32.09, 185.05, *p* = 0.01). The ICC for Model 3 was 27.6%, indicating that these variables accounted for 25.8% of the variation in prices.

### 3.4 Multilevel characteristics and prices for pelvis CT scan

In Model 0, the ICC for pelvis CT was 50.9%. Prices differed by hospital ownership, type, and number of beds (Model 1), as well as county‐level Census region, percent uninsured, unemployment, educational attainment, and life expectancy (Model 2; all *p* < 0.05). In Model 3, prices were higher for hospitals that were proprietary or volunteer versus government ownership (proprietary: *β* = 1483.30, 95% CI: 1158.51, 1808.09, *p* < 0.001; volunteer: *β* = 410.38, 95% CI: 135.29, 685.47, *p* < 0.01) and with more beds (*β* = 48.32, 95% CI: 6.20, 90.43, *p* = 0.02), and for hospitals in counties in the South versus Midwest region (*β* = 320.85, 95% CI: 20.68, 621.02, *p* = 0.04), with higher percent uninsured (*β* = 98.51, 95% CI: 72.08, 124.94, *p* < 0.001), with higher unemployment (*β* = 22.78, 95% CI: 8.90, 36.65, *p* < 0.01), and with higher life expectancy (*β* = 86.03, 95% CI: 39.91, 132.16, *p* < 0.001) (Table 1). The ICC for Model 3 was 41.5%, indicating that these variables accounted for 18.4% of the variation in prices.

### 3.5 Multilevel characteristics and commercial insurance prices

Commercial insurance prices were generally lower for hospitals in urban counties (Table S3). Commercial insurance prices also varied by hospital compliance score, with a negative association observed for abdominal ultrasound but positive associations observed for the remaining procedures. The ICCs for each procedure were moderate (abdominal ultrasound: ICC = 29.3%; diagnostic colonoscopy: ICC = 18.8%; brain MRI: ICC = 38.6%; pelvis CT: ICC = 30.0%).

## 4 DISCUSSION

This study examined the relationship between hospital and county characteristics and healthcare prices for four procedures commonly used in cancer care. In terms of hospital characteristics, prices were higher at proprietary hospitals compared to government‐owned hospitals for all four procedures; abdominal ultrasound prices were lower at critical access hospitals compared to acute care hospitals; and prices for diagnostic colonoscopy, brain MRI, and pelvis CT were higher at hospitals with more hospital beds. Such findings are consistent with literature indicating that larger, for‐profit hospitals charge higher prices than smaller, non‐profit hospitals.^22^, ^23^, ^24^ In terms of county characteristics, prices differed by region and health insurance coverage. The ICCs suggest that county location accounted for a substantial proportion of variation in prices.

Geographic location of the hospitals helped explain pricing, with elevated prices for abdominal ultrasound, brain MRI, and pelvis CT in the South. Many hospitals with the highest price markups are located in Southern states, which motivates calls for federal and state policies to limit the charge‐to‐cost ratios.^25^ Further, we found that county‐level social determinants of health were associated with procedure prices. Specifically, list prices were positively associated with the percent of the population that was uninsured, which supports prior literature suggesting that hospitals raise prices to subsidize the risk of uncompensated care delivered to patients without health insurance.^22^ It is important to note that list price is often different from the actual out‐of‐pocket costs incurred by uninsured patients, which can be substantially lower. One study reported that most of the hospital revenue from care for the uninsured was from a subset of patients who paid full or near‐full list price.^26^

Overall, our study did not find consistent differences in prices by metropolitan status or density of PCPs, which differs from previous studies demonstrating that providers in low‐access counties have greater negotiation power, resulting in higher prices for primary care visits.^5^ However, our results suggest that this relationship does not hold true for the four cancer‐related procedures included in this study.

List prices differed from commercial insurance prices, even after accounting for county‐ and hospital‐characteristics, which is consistent with previous findings.^2^, ^27^ Procedure prices for commercial insurance were generally related to hospital compliance score and county‐level urbanicity. Price variation for commercial insurance may relate to negotiations between insurers and hospitals, or they may indicate that the markets are not operating efficiently.^28^ In general, as we observed in this study, greater market power among providers (e.g., in rural counties) can lead to higher prices for commercial insurers.^28^

These findings have implications for interventions and policies focused on healthcare costs and accessibility. The CMS policy requiring hospitals to publish procedure prices was implemented in 2021 to empower patients and energize market competition.^10^ However, in low‐access counties (including rural counties), patients have fewer options for accessing healthcare; as a result, competition is limited. Complementary price transparency policies could also impact costs for other aspects of care. For example, a recent study showed that prices for commonly‐used chemotherapeutics at cancer centers were 188–634% higher than the estimated costs to manufacturers.^29^ It is important to remember that healthcare prices are not the same as costs incurred to insurers or out‐of‐pocket costs for patients,^30^ so additional research is needed to understand these interrelationships and how patients can understand and navigate costs. Additional interventions and policies are needed to ensure healthcare accessibility and minimize costs, including efforts to ensure prices are available to patients in a digestible and consumer‐friendly format. In addition, efforts are needed to reduce the burden of cancer diagnosis and treatment, particularly for patients in certain geographic areas, e.g., in the South and in counties with low health insurance coverage.^31^, ^32^ Overall, additional research is needed on the correlation between listed prices and incurred costs.

These findings have implications for cancer inequities and for clinical practice with at‐risk populations. Low‐resource patients may delay, refuse, or discontinue cancer screening and treatment.^33^ For example, low‐income women may delay cervical cancer screening because of high screening costs.^34^ Clinicians' role in helping patients manage treatment costs include screening patients for financial toxicity, discussing healthcare costs and concerns, and working with an interdisciplinary team that provides supportive care services.^35^ Patient navigators, nurse case managers, and social workers can assist patients in understanding healthcare costs and connecting patients with available resources.^35^

In terms of study strengths, our analysis leveraged a large dataset with coverage across 2004 hospitals and 1319 counties in the U.S. We conducted a comprehensive assessment of several multilevel characteristics theoretically and empirically related to hospital prices, across four illustrative cancer‐related healthcare procedures, to demonstrate the breadth and reliability of our findings. In particular, analyzing differences in list prices and commercial insurance prices extended on prior literature (often focused on Medicare/Medicaid prices) and highlighted differences in multilevel characteristics associated with prices across health insurance types.

In terms of study limitations, the price dataset is limited to hospitals that published publicly‐available, machine‐readable data on prices, and therefore is incomplete. Thus, hospitals included in the dataset may differ from excluded hospitals in systematic ways. Further, data availability limited our analysis to four cancer‐related procedures; clearly, additional procedures are administered for cancer diagnosis and treatment, and the included procedures could be used for non‐cancer purposes. Future research should examine additional cancer‐related procedures to extend our findings. Excluded variables may be important correlates of healthcare prices, which could bias the study findings. However, our models included random county‐level intercepts, which serve to minimize the influence of unmeasured county‐level characteristics.

## 5 CONCLUSIONS

Prices for cancer‐related hospital procedures varied substantially by hospital, county, and health insurance type. County‐level social determinants of health (e.g., levels of health insurance coverage and unemployment, metropolitan status) were consistently associated with procedure prices, helping to explain geographic variation in the financial burden of cancer care. Patients from lower resource communities may be charged the highest prices for cancer‐related healthcare procedures. Further research is needed to determine whether price transparency programs drive reductions in healthcare costs and spending and ultimately impact cancer outcomes. Policy and programmatic interventions to alleviate financial burden are needed to ensure that patients can access the healthcare they need to effectively diagnose and treat cancer.

## AUTHOR CONTRIBUTIONS

**Jennifer Moss:** Conceptualization (equal); formal analysis (equal); investigation (lead); methodology (lead); project administration (lead); resources (equal); software (equal); supervision (lead); writing – original draft (lead); writing – review and editing (equal). **Savanna Ledford:** Investigation (equal); validation (equal); visualization (equal); writing – original draft (equal); writing – review and editing (equal). **Veronica Bernacchi:** Validation (equal); visualization (equal); writing – original draft (equal); writing – review and editing (equal). **Chan Shen:** Conceptualization (equal); data curation (lead); methodology (equal); resources (equal); software (equal); visualization (lead); writing – original draft (equal); writing – review and editing (equal).

## FUNDING INFORMATION

No external funding was received for this study. Moss's time was supported by K22 CA225705 (PI: Moss). Bernacchi's time was supported by the Presidential Post‐doctoral Fellowship Program.

## CONFLICT OF INTEREST STATEMENT

The authors have no conflicts of interest to disclose.

## PRECIS

Prices for cancer‐related hospital procedures vary substantially across counties, with higher prices observed in counties with a higher percentage of uninsured population. Variation in healthcare pricing can contribute to disparities in treatment costs and other cancer outcomes.

## Supporting information


Data S1:

## Data Availability

The data that support the findings of this study are available from Turquoise Health. Restrictions apply to the availability of these data, which were used under license for this study. Data are available at http://www.turquoise.health.

## References

[1] Congress of the United States Congressional Budget Office . Geographic variation in health care spending. https://www.cbo.gov/sites/default/files/cbofiles/ftpdocs/89xx/doc8972/02‐15‐geoghealth.pdf 2008.

[2] Shen C , Moss JL . Large variations in hospital pricing for standard procedures revealed. BMC Res Notes. 2022;15(1):129. 35382890 10.1186/s13104-022-06014-2 PMC8981177

[3] Wu SJ , Sylwestrzak G , Shah C , DeVries A . Price transparency for MRIs increased use of less costly providers and triggered provider competition. Health Aff (Millwood). 2014;33(8):1391‐1398. 25092841 10.1377/hlthaff.2014.0168

[4] Newhouse JP , Garber AM . Geographic variation in health care spending in the United States: insights from an Institute of Medicine report. JAMA. 2013;310(12):1227‐1228. 24008265 10.1001/jama.2013.278139

[5] Williams D Jr , Holmes M . Rural health care costs: are they higher and why might they differ from urban health care costs? N C Med J. 2018;79(1):51‐55. 29439106 10.18043/ncm.79.1.51

[6] Riley T , Cousart C . Health Care Is Local: Impact of Income and Geography on Premiums and Premium Support. The National Academy for State Health Policy. http://nashp.org/wp‐content/uploads/2017/06/Health‐Care‐is‐Local1.pdf; 2017.

[7] Montero A , Kearney A , Hamel L , Brodie M . Americans' Challenges with Health Care Costs. KFF. https://www.kff.org/health‐costs/issue‐brief/americans‐challenges‐with‐health‐care‐costs/; 2021.

[8] Lentz R , Benson AB 3rd , Kircher S . Financial toxicity in cancer care: prevalence, causes, consequences, and reduction strategies. J Surg Oncol. 2019;120(1):85‐92. 30650186 10.1002/jso.25374

[9] Yabroff KR , Mariotto A , Tangka F , et al. Annual report to the nation on the status of cancer, part 2: patient economic burden associated with cancer care. J Natl Cancer Inst. 2021;113:1670‐1682. 34698839 10.1093/jnci/djab192 PMC9891103

[10] Centers for Medicare & Medicaid Services . Hospital price transparency. https://www.cms.gov/hospital‐price‐transparency 2021.

[11] Wheeler C , Taylor R . New Year, New CMS Price Transparency Rule For Hospitals. Health Affairs Blog 2021.

[12] Turquoise Health . Price transparency data for researchers. https://turquoise.health/researchers 2022.

[13] Gray BH . Changes in the ownership, control, and configuration of health care services. For‐Profit Enterprise in Health Care. National Academies Press (US); 1986. 25032434

[14] U.S. Department of Health and Human Services . Area Health Resources Files. https://data.hrsa.gov/topics/health‐workforce/ahrf 2021.

[15] U.S. Census Bureau . Census Regions and Divisions of the United States. https://www2.census.gov/geo/pdfs/maps‐data/maps/reference/us_regdiv.pdf

[16] U.S. Department of Agriculture . Rural‐Urban Continuum Codes: Overview. http://www.ers.usda.gov/data‐products/rural‐urban‐continuum‐codes.aspx 2019.

[17] County Health Rankings & Roadmaps . Explore Health Rankings. https://www.countyhealthrankings.org/explore‐health‐rankings 2021.

[18] National Institute of Standards and Technology . Current FIPS. https://www.nist.gov/itl/current‐fips 2018.

[19] Blakely TA , Subramanian SV . Multilevel studies. In: Oakes JM , Kaufman JS , eds. Methods in Social Epidemiology. Jossey‐Bass; 2006:316‐340.

[20] Subramanian SV . The relevance of multilevel statistical methods for identifying causal neighborhood effects. Soc Sci Med. 2004;58(10):1961‐1967. 15020011 10.1016/S0277-9536(03)00415-5

[21] Bell BA , Ene M , Smiley W , Schoeneberger JA . A multilevel model primer using SAS PROC MIXED. SAS Global Forum 2013.

[22] Bai G , Anderson GF . US hospitals are still using chargemaster markups to maximize revenues. Health Aff (Millwood). 2016;35(9):1658‐1664. 27605648 10.1377/hlthaff.2016.0093

[23] Batty M , Ippolito B . Mystery of the chargemaster: examining the role of hospital list prices in what patients actually pay. Health Aff (Millwood). 2017;36(4):689‐696. 28373335 10.1377/hlthaff.2016.0986

[24] Cooper Z , Craig SV , Gaynor M , Van Reenen J . The price AIN'T right? Hospital prices and health spending on the privately insured. Q J Econ. 2019;134(1):51‐107. 32981974 10.1093/qje/qjy020 PMC7517591

[25] Bai G , Anderson GF . Extreme markup: the fifty US hospitals with the highest charge‐to‐cost ratios. Health Aff (Millwood). 2015;34(6):922‐928. 26056196 10.1377/hlthaff.2014.1414

[26] Batty M , Ippolito B . Financial incentives, hospital care, and health outcomes: evidence from fair pricing laws. Am Econ J Econ Pol. 2017;9(2):28‐56.

[27] White C , Whaley CM . Prices paid to hospitals by private health plans are high relative to Medicare and vary widely: findings from an employer‐led transparency initiative. Rand Health Q. 2021;9(2):5. 34484877 10.7249/rhq-09-02-05 PMC8383843

[28] Congressional Budget Office . The prices that commercial health insurers and Medicare pay for hospitals' and physicians' services. https://www.cbo.gov/system/files/2022‐01/57422‐medical‐prices.pdf 2022.

[29] Xiao R , Ross JS , Gross CP , et al. Hospital‐administered cancer therapy prices for patients with private health insurance. JAMA Intern Med. 2022;182(6):603‐611. 35435948 10.1001/jamainternmed.2022.1022 PMC9016607

[30] White C , Reschovsky JD , Bond AM . Understanding differences between high‐ and low‐price hospitals: implications for efforts to rein in costs. Health Aff (Millwood). 2014;33(2):324‐331. 24476706 10.1377/hlthaff.2013.0747

[31] Hunter WG , Zhang CZ , Hesson A , et al. What strategies do physicians and patients discuss to reduce out‐of‐pocket costs? Analysis of cost‐saving strategies in 1,755 outpatient clinic visits. Med Decis Mak. 2016;36(7):900‐910. 10.1177/0272989X15626384 PMC4955629 26785714

[32] Yezefski T , Steelquist J , Watabayashi K , Sherman D , Shankaran V . Impact of trained oncology financial navigators on patient out‐of‐pocket spending. Am J Manag Care. 2018;24(5 Suppl):S74‐S79. 29620814

[33] Abrams HR , Durbin S , Huang CX , et al. Financial toxicity in cancer care: origins, impact, and solutions. Transl Behav Med. 2021;11(11):2043‐2054. 34850932 10.1093/tbm/ibab091

[34] Biddell CB , Spees LP , Smith JS , et al. Perceived financial barriers to cervical cancer screening and associated cost burden among low‐income, under‐screened women. J Womens Health (Larchmt). 2021;30(9):1243‐1252. 33851854 10.1089/jwh.2020.8807 PMC8558088

[35] Carrera PM , Kantarjian HM , Blinder VS . The financial burden and distress of patients with cancer: understanding and stepping‐up action on the financial toxicity of cancer treatment. CA Cancer J Clin. 2018;68(2):153‐165. 29338071 10.3322/caac.21443 PMC6652174

