# Supplementary material for: Hospital‐ and county‐level characteristics explain geographic variability in prices of cancer‐related procedures: Implications for policy and interventions
Source: Cancer Med. 2023 Dec 22;13(1):e6792. doi: 10.1002/cam4.6792 (PMC10807617; doi:10.1002/cam4.6792)
Supplement: Supplementary file 1 — Data S1: [file CAM4-13-e6792-s001.docx]

**Supplementary Table S1.** Characteristics of hospitals and counties in analytic sample.

| **Hospitals (*n*=2,004)** | | | |
| --- | --- | --- | --- |
|  | *n* | % |  |
| Ownership |  |  |  |
| Government | 200 | 11.6 |  |
| Physician | 14 | 0.8 |  |
| Proprietary | 289 | 16.7 |  |
| Volunteer | 1223 | 70.9 |  |
| Type |  |  |  |
| Acute care | 1372 | 79.5 |  |
| Critical access | 354 | 20.5 |  |
|  |  |  |  |
|  | median | mean | 95% CI |
| Quality rating | 4.0 | 4.1 | (4.1-4.2) |
| Number of beds (100s) | 1.3 | 2.0 | (1.9-2.2) |
|  |  |  |  |
| **Counties (*n*=1,319)** | | | |
|  | *n* | % |  |
| Census region |  |  |  |
| Midwest | 473 | 36.2 |  |
| Northeast | 137 | 10.5 |  |
| South | 557 | 42.6 |  |
| West | 141 | 10.8 |  |
| Metropolitan status |  |  |  |
| Non-metropolitan/Rural | 648 | 49.1 |  |
| Metropolitan/Urban | 671 | 50.9 |  |
|  |  |  |  |
|  | median | mean | 95% CI |
| County population (1000s) | 54.4 | 195.1 | (168.7-221.6) |
| Density of PCPs (number/100,000 population) | 54.1 | 60.6 | (58.6-62.6) |
| % without health insurance | 9.7 | 10.8 | (10.5-11.0) |
| % below the federal poverty level | 15.1 | 16.4 | (15.9-16.8) |
| % unemployed^1^ | 3.9 | 4.1 | (4.0-4.1) |
| % with bachelor's degree^2^ | 21.5 | 25.4 | (24.7-26.1) |
| Estimated life expectancy (years) | 77.8 | 77.8 | (77.6-77.9) |

*Note.* CI=confidence interval; PCPs=primary care physicians.

^1^Among population, ages 16+, in the civilian workforce.

^2^Among adults ages 25+.

**Supplementary Table S2.** Distribution of prices (in dollars) for four cancer-related procedures, list prices and prices for commercial insurance (*n*=2,004 hospitals).

| **Procedure** | **Median** | **Mean** | **95% CI** | **CV** |
| --- | --- | --- | --- | --- |
| Abdominal ultrasound |  |  |  |  |
| List | 1,050 | 1,267 | (1,200-1,335) | 101 |
| Commercial insurance | 531 | 669 | (659-679) | 134 |
| Diagnostic colonoscopy |  |  |  |  |
| List | 2,694 | 4,780 | (4,185-5,375) | 186 |
| Commercial insurance | 1,933 | 2,525 | (2,483-2,567) | 123 |
| MRI scan of the brain |  |  |  |  |
| List | 4,390 | 4,988 | (4,832-5,144) | 59 |
| Commercial insurance | 1,905 | 2,497 | (2,471-2,523) | 94 |
| Pelvis CT scan with contrast |  |  |  |  |
| List | 2,483 | 2,883 | (2,787-2,979) | 62 |
| Commercial insurance | 1,190 | 1,450 | (1,428-1,472) | 132 |

*Note.* CI=confidence interval; CV=coefficient of variation; MRI=magnetic resonance imaging; CT=computerized tomography.

**Supplementary Table S3.** Multilevel relationships between hospital- and county-level characteristics and commercial insurance prices for selected procedures, across hospitals.

|  | **Abdominal ultrasound (*n*=914 hospitals)** | | | **Diagnostic colonoscopy (*n*=761 hospitals)** | | | **MRI of the brain  (*n*=1,013 hospitals)** | | | **Pelvic CT scan with contrast (*n*=881 hospitals)** | | |
| --- | --- | --- | --- | --- | --- | --- | --- | --- | --- | --- | --- | --- |
|  | beta | *SE* | *p* | beta | *SE* | *p* | beta | *SE* | *p* | beta | *SE* | *p* |
| *Level 1: Hospital-level characteristics* |  |  |  |  |  |  |  |  |  |  |  |  |
| Ownership |  |  |  |  |  |  |  |  |  |  |  |  |
| Government | (ref) | | | (ref) | | | (ref) | | | (ref) | | |
| Physician | -1.78 | 142.77 | 0.99 | -377.61 | 425.18 | 0.37 | -122.24 | 277.47 | 0.66 | -364.05 | 432.87 | 0.40 |
| Proprietary | -93.83 | 33.88 | <.01 | 230.59 | 154.42 | 0.14 | 7.04 | 81.20 | 0.93 | -91.42 | 83.41 | 0.27 |
| Volunteer | 201.11 | 29.12 | <.001 | -135.04 | 122.90 | 0.27 | 359.47 | 71.48 | <.001 | -92.19 | 73.77 | 0.21 |
| Type |  |  |  |  |  |  |  |  |  |  |  |  |
| Acute care | (ref) | | | (ref) | | | (ref) | | | (ref) | | |
| Critical access | -137.88 | 31.67 | <.001 | 27.24 | 129.90 | 0.83 | 82.59 | 77.81 | 0.29 | 368.18 | 78.28 | <.001 |
| Compliance score | -107.03 | 17.23 | <.001 | 168.75 | 76.35 | 0.03 | 97.30 | 41.71 | 0.02 | 118.93 | 44.01 | 0.01 |
| Number of beds (100s) | -4.37 | 3.80 | 0.25 | 32.38 | 17.70 | 0.07 | -7.56 | 8.98 | 0.40 | -8.19 | 9.41 | 0.38 |
|  |  |  |  |  |  |  |  |  |  |  |  |  |
| *Level 2: County-level characteristics* |  |  |  |  |  |  |  |  |  |  |  |  |
| Census region |  |  |  |  |  |  |  |  |  |  |  |  |
| Midwest | (ref) | | | (ref) | | | (ref) | | | (ref) | | |
| Northeast | -143.39 | 59.82 | 0.02 | -355.07 | 204.56 | 0.08 | -390.18 | 167.95 | 0.02 | -275.86 | 150.00 | 0.07 |
| South | 129.78 | 54.75 | 0.02 | -249.22 | 184.92 | 0.18 | 288.83 | 148.29 | 0.05 | -29.58 | 130.76 | 0.82 |
| West | 85.58 | 60.51 | 0.16 | 422.79 | 208.01 | 0.04 | 577.48 | 169.43 | <.001 | 280.22 | 147.19 | 0.06 |
| Metropolitan status |  |  |  |  |  |  |  |  |  |  |  |  |
| Non-metropolitan/Rural | (ref) | | | (ref) | | | (ref) | | | (ref) | | |
| Metropolitan/Urban | -201.78 | 42.20 | <.001 | -151.16 | 145.27 | 0.30 | -584.17 | 114.70 | <.001 | -287.86 | 102.60 | 0.01 |
| County population (1000s) | -0.01 | 0.03 | 0.85 | -0.21 | 0.10 | 0.03 | -0.15 | 0.09 | 0.10 | -0.10 | 0.08 | 0.21 |
| Density of PCPs (number/100,000 population) | 0.47 | 0.62 | 0.45 | -1.02 | 2.07 | 0.62 | 1.68 | 1.57 | 0.28 | 0.15 | 1.52 | 0.92 |
| % without health insurance | 1.18 | 4.98 | 0.81 | 14.56 | 16.81 | 0.39 | -13.18 | 13.79 | 0.34 | 3.00 | 12.02 | 0.80 |
| % below the federal poverty level | 0.24 | 1.26 | 0.85 | -6.45 | 5.15 | 0.21 | 4.72 | 3.04 | 0.12 | 2.83 | 3.20 | 0.38 |
| % unemployed^1^ | -8.79 | 1.67 | <.001 | -13.38 | 6.75 | 0.05 | 2.10 | 4.02 | 0.60 | 0.86 | 4.30 | 0.84 |
| % with bachelor's degree^2^ | 0.06 | 0.67 | 0.93 | -2.69 | 3.06 | 0.38 | 7.33 | 1.65 | <.001 | 4.31 | 1.74 | 0.01 |
| Estimated life expectancy (years) | -12.00 | 8.31 | 0.15 | -34.81 | 28.49 | 0.22 | 39.87 | 22.61 | 0.08 | 1.73 | 19.98 | 0.93 |
|  |  |  |  |  |  |  |  |  |  |  |  |  |
| ICC (empty model) | 26.3% |  |  | 20.5% |  |  | 37.5% |  |  | 25.3% |  |  |
| ICC (full model) | 29.3% |  |  | 18.8% |  |  | 38.6% |  |  | 30.0% |  |  |

*Note.* MRI=magnetic resonance imaging; CT=computerized tomography; SE=standard error; ref=reference; PCP=primary care provider; ICC=intraclass correlation.

^1^Among population, ages 16+, in the civilian workforce.

^2^Among adults 25+ years.
